# Targeting Mitochondrial STAT3 with the Novel Phospho-Valproic Acid (MDC-1112) Inhibits Pancreatic Cancer Growth in Mice

**DOI:** 10.1371/journal.pone.0061532

**Published:** 2013-05-01

**Authors:** Gerardo G. Mackenzie, Liqun Huang, Ninche Alston, Nengtai Ouyang, Kvetoslava Vrankova, George Mattheolabakis, Panayiotis P. Constantinides, Basil Rigas

**Affiliations:** 1 Division of Cancer Prevention, Department of Medicine, Stony Brook University, Stony Brook, New York, United States of America; 2 Medicon Pharmaceuticals, Inc, Stony Brook, New York, United States of America; Technische Universität München, Germany

## Abstract

New agents are needed to treat pancreatic cancer, one of the most lethal human malignancies. We synthesized phospho-valproic acid, a novel valproic acid derivative, (P-V; MDC-1112) and evaluated its efficacy in the control of pancreatic cancer. P-V inhibited the growth of human pancreatic cancer xenografts in mice by 60%–97%, and 100% when combined with cimetidine. The dominant molecular target of P-V was STAT3. P-V inhibited the phosphorylation of JAK2 and Src, and the Hsp90-STAT3 association, suppressing the activating phosphorylation of STAT3, which in turn reduced the expression of STAT3-dependent proteins Bcl-x_L_, Mcl-1 and survivin. P-V also reduced STAT3 levels in the mitochondria by preventing its translocation from the cytosol, and enhanced the mitochondrial levels of reactive oxygen species, which triggered apoptosis. Inhibition of mitochondrial STAT3 by P-V was required for its anticancer effect; mitochondrial STAT3 overexpression rescued animals from the tumor growth inhibition by P-V. Our results indicate that P-V is a promising candidate drug against pancreatic cancer and establish mitochondrial STAT3 as its key molecular target.

## Introduction

Pancreatic cancer (PC), referred to as “the dismal disease” because of its aggressive nature and high mortality, is one of the most devastating malignancies worldwide, being often fatal within 6 months [1]. The high mortality of PC is ascribed to late diagnosis, rapid disease progression and resistance to chemotherapy. Because of the disappointing performance of current treatments, there is an urgent need to identify novel agents for its treatment.

The Signal Transducer and Activator of Transcription 3 (STAT3) transcription factor plays a significant role in the pathogenesis of PC, being associated with malignant tumor initiation, transformation and progression [2], [3]. Besides its established nuclear transcriptional role, STAT3 plays a distinct role in the mitochondria, where it supports Ras-dependent malignant transformation [4], and ensures the optimal function of the electron transport chain [5]. Because it regulates several pathways important in tumorigenesis [6], STAT3 is recognized as a potential drug target for PC [7].

**Figure 1:**
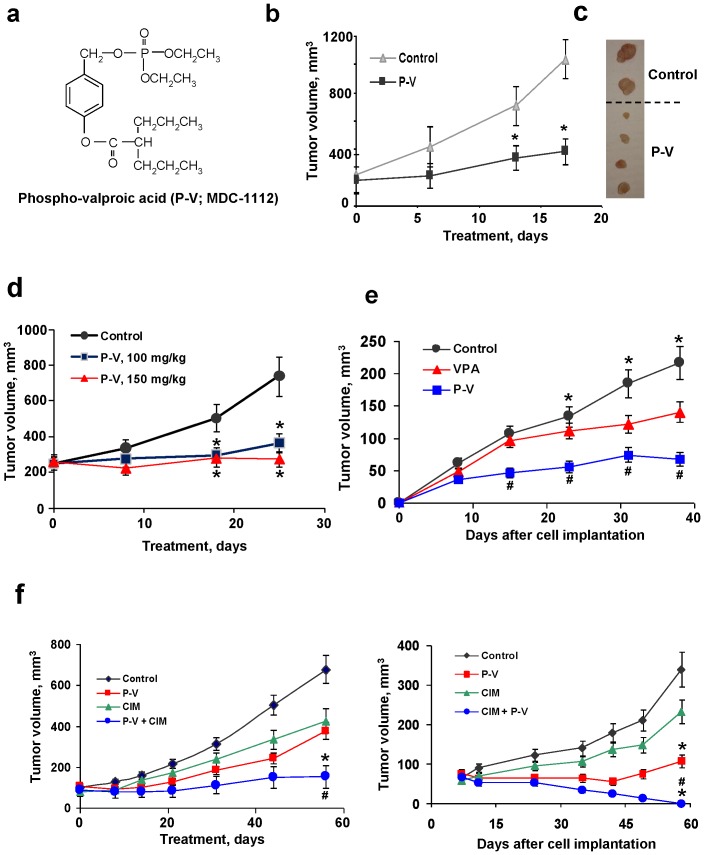
P-V inhibits the growth of human PC xenografts: synergy with cimetidine. (**A**) The chemical structure of P-V (MDC-1112). (**B**) BxPC-3 tumor volume growth of control (▴) and P-V 50 mg/kg/d (▪) treated mice. *p<0.01 *vs*. control group. (**C**) Images of representative tumors from control and P-V-treated mice. (**D**) MIA PaCa-2 tumor volume growth of control (•), 100 mg/kg (▪) and 150 mg/kg (▴) P-V-treated mice. *p<0.01 *vs*. control. (**E**) BxPC-3 tumor volume growth of control, P-V 50 mg/kg/d and VPA 250 mg/kg/d treated mice. *p<0.01 *vs*. P-V and VPA groups; ^#^ *p*<0.01 *vs*. VPA group. (**F**) Cimetidine (CIM) synergizes with P-V to inhibit the growth of human PC xenografts. Nude mice bearing MIA PaCa-2 (*left*) or BxPC-3 (*right*) xenografts were treated with P-V 50 mg/kg, CIM 100 mg/kg, or both. *p<0.01 *vs*. control; ^#^ *p*<0.05 *vs*. P-V or CIM groups.

Rational drug development requires, among others, exploiting the properties of putative molecular targets. We have identified phospho-valproic acid (P-V; MDC-1112; Fig. 1A), a novel valproic acid (VPA) derivative, as a potent STAT3 inhibitor. This agent has been synthesized based on a general approach where a specific chemical modification of known drugs enhances their desired anticancer properties, primarily their efficacy. The anticancer properties of VPA, a branched short-chain fatty acid widely used as an antiepileptic drug, are under study, especially since it was identified as a histone deacetylation (HDAC) inhibitor [8]–[10]. Ongoing trials with VPA show encouraging results for several human malignancies [11].

Herein, we established P-V as a potent agent for PC control, identified cimetidine as a strong combination partner and outlined its mechanism of action, which involved the inhibition of STAT3 at the mitochondrial level.

## Materials and Methods

### Reagents - Cell culture

P-V and phospho-aspirin (MDC-43) were a gift from Medicon Pharmaceuticals Inc. (Stony Brook, NY). Cimetidine and VPA were purchased from Sigma (St Louis, MO). Dihydroethidium (DHE), 2′,7′-dichlorodihydrofluorecein diacetate (DCFDA), MitoSOX Red and Annexin V were purchased from Invitrogen. All general solvents and reagents were of HPLC grade or the highest grade commercially available.

Human pancreatic (AsPC-1, BxPC-3, Capan-2, CFPAC-1, HPAF-II, Panc-1 and MIA PaCa-2), breast (MCF-7 and MDA-MB 231) and colon (HT-29, and SW-480) cells were grown as suggested by ATCC (Manassas, VA). All the cell lines were passaged in our laboratory for less than 6 months after receipt. The mitochondria-less (ρ^0^) derivatives of BxPC-3 cells were obtained by incubating cells with 200 ng/ml ethidium bromide and 50 µg/ml uridine for 8 weeks [12].

Cell growth was determined using the MTT assay. Cell proliferation was assayed by 5-bromo-2′-deoxyuridine (BrdU) incorporation; apoptosis and necrosis by staining with Annexin V-FITC and propidium iodide (PI) and analyzing the fluorescence intensities by FACScaliber (BD Bioscience); and cell cycle by flow cytometry, all as described [13].

### Antibody microarray

The Kinex Antibody Microarray analyses were performed on protein lysates obtained from BxPC-3 cells treated with or without P-V for 2 h following the instructions from Kinexus (www.kinexus.ca).

### Determination of ROS levels

Cells were treated with test agents for 1 h, stained with 10 µM DCFDA, 10 µM DHE or 10 µM MitoSOX Red for 30 min at 37°C and fluorescence intensity was analyzed by FACScaliber.

### Determination of mitochondrial O_2_^−^ by fluorescence microscopy

Cells seeded overnight in glass bottom culture dishes (MatTek, Ashland, MA) were treated with P-V and assayed by fluorescence microscopy [14].

### Determination of mitochondrial membrane potential

The mitochondrial membrane potential (ΔΨm) was determined by flow cytometry using the JC-1 cationic dye [13].

### Determination of the mitochondrial electron transport chain complex I activity

The activity of the mitochondrial electron transport chain complex I was determined in mitochondrial extracts from MIA PaCa-2 cells incubated with P-V 1.5×IC_50_ for 1 h, following the manufacturer's instructions (MitoSciences, Eugene, OR).

### Mitochondrial import assay

The *in vitro* mitochondrial import assays were performed as described [15] (details in Methods S1).

### Determination of HDAC activity

The HDAC activity was determined using a colorimetric-based assay. Briefly, Panc-1 cells were treated with P-V, VPA or the HDAC inhibitor trichostatin A for 3 h and the HDAC activity was then evaluated following the manufacturer's instructions (BioVision; Milpitas, CA).

### Western blot analysis

Whole cell fractions were obtained as described [13]. Cytosolic and mitochondrial fractions were obtained using the Mitochondria Fraction Isolation Kit (Pierce; Rockford, IL). Western blots were performed as described [13].

### STAT3 gene silencing

Cells were transfected with 100 nmol/L STAT3 small interfering RNA (siRNA) or nonspecific control siRNA (details in Methods S1).

### STAT3 overexpression

Cells were transiently or stably transfected with the STAT3 expression plasmid (STAT3 cDNA; OriGene, Rockville, MD; details in Methods S1). For the *in vivo* studies, MIA PaCa-2 cells with silenced STAT3, were transfected with STAT3 Y705F Flag pRc/CMV plasmid [16], a gift of James Darnell (Rockefeller University, NY) (Addgene plasmid # 8709). On the other hand, AsPC-1 cells, with silenced STAT3, were transfected with the MLS-STAT3 plasmid [17], a gift of Dr. Andrew Larner.

### Animal studies

All animal experiments were approved by our Institutional Animal Care and Use Committee of Stony Brook University.

### Nude mice xenograft studies

#### Chemotherapy protocol

Female BALB/c nude mice (5–6 weeks-old; Charles River, Wilmington, MA) were subcutaneously injected with 1.5×10^6^ BxPC-3 or MIA PaCa-2 cells in 100 µl PBS into each flank. When the tumors reached 200 mm^3^, mice (n = 10/group) were randomized into groups receiving corn oil (control), or P-V (50, 100 or 150 mg/kg) in corn oil given orally 5×/wk.

#### Chemopreventive protocol

One week prior to implanting BxPC-3 cells, mice were divided into three groups (control, P-V, and VPA; n = 7/group) and started on P-V 50 mg/kg/d or VPA 250 mg/kg/d orally by gavage for 7 weeks; these doses represent 25% of their respective maximum tolerated doses (MTD).

#### Combination study

Female NCr-nude mice (5–6 weeks-old; Taconic, Hudson, NY) were subcutaneously inoculated into each flank with 1.5×10^6^ BxPC-3 or MIA PaCa-2 cells in 100 µL PBS and randomized into four groups (n = 7/group) receiving 5×/wk for 58 days: vehicle (corn oil); 50 mg/kg P-V in corn oil by oral gavage; 100 mg/kg cimetidine in PBS i.p.; and P-V plus cimetidine as above.

### Orthotopic pancreatic xenograft study

Parent, STAT3-overexpressing or STAT3^Y705F^-overexpressing MIA PaCa-2 cells, or parent and MLS-STAT3 overexpressing AsPC-1 cells (1×10^6^ in 30 µL PBS) were injected into the parenchyma of the pancreas with a 27-gauge hypodermic needle. Five days later, mice were randomized into two groups (n = 8/group) and treated with vehicle (corn oil) or P-V 150 mg/kg 5×/wk orally by gavage for 18 or 38 days.

### Immunohistochemistry

Staining for Ki-67, Amylase, STAT3, p-STAT3^Ser727^, p-STAT3^Tyr705^, Mcl-1 and Bcl-2 was performed as described.[18] Apoptosis was determined immunohistochemically by the terminal deoxynucleotidyl transferase-mediated deoxyuridine triphosphate-biotin nick end-labeling (TUNEL) assay [14]. See for more details.

### Statistical analyses

Results from at least three independent experiments were expressed as *mean*±*SEM* and analyzed by one-factor analysis of variance followed by Tukey test for multiple comparisons. *P*<0.05 was statistically significant.

## Results

### P-V inhibits the growth of human PC xenografts in mice: Synergy with cimetidine

To assess the chemotherapeutic potential of P-V, we employed both orthotopic and heterotopic (subcutaneous) PC xenograft models in nude mice using two human PC cell lines differing in their *Kras* status, BxPC-3 (wild-type *Kras*) and MIA PaCa-2 (mutant *Kras*) (Fig. 1B–D). P-V 50 mg/kg/d inhibited the growth of BxPC-3 subcutaneous xenografts, reducing on day 17 the tumor volume by 75.4% compared to control (p<0.01). At doses of 100 and 150 mg/kg/x25d, P-V inhibited the growth of subcutaneous MIA PaCa-2 xenografts by 76.6% and 96.9%, respectively (p<0.01). A somewhat lower inhibitory effect was noted in orthotopic MIA PaCa-2 xenografts in mice treated with P-V 150 mg/kg/d starting 5 days post-implantation and continuing for 38 days (60% inhibition at sacrifice; p<0.001; Fig. S1).

We also compared the anticancer effects of P-V and VPA, its parent compound, by treating nude mice bearing subcutaneous BxPC-3 xenografts with P-V 50 mg/kg/d and VPA 250 mg/kg/d; these doses represent 25% of their respective MTDs. We followed a prevention protocol, i.e. treatment started 1 wk prior to BxPC-3 implantation. On day 38 post-implantation, compared to controls P-V reduced xenograft tumor volume by 68% whereas VPA reduced it by 34% (p<0.01 for both), with the difference between the two being significant (p<0.05; Fig. 1E).

Given the aggressive nature of PC, we explored whether P-V could be successfully combined with other agents. We screened several compounds in vitro. Isobolograms established synergy between P-V and 5-FU, GABA, arsenic trioxide and cimetidine (Fig. S2); all are reported to have some effect against PC [19]–[22]. While 5-FU failed to synergize with P-V in vivo (Fig. S3), cimetidine, a histamine-2 blocker, had a dramatic effect on the growth of PC xenografts when combined with P-V (Fig. 1F). Administered under a treatment protocol to mice with MIA PaCa-2 xenografts, the combination essentially produced tumor stasis; each compound alone had only a partial effect (<45% inhibition at the end of the study). Administered under a prevention protocol to mice with BxPC-3 xenografts, cimetidine plus P-V eliminated all tumors in all animals by day 58, in contrast to each compound alone (31% inhibition by cimetidine and 68% by P-V).

P-V reduced tumor growth through its cytokinetic effect. For example, in the study of heterotopic BxPC-3 xenografts, P-V inhibited cell proliferation by 49% and increased apoptosis by 78% compared to controls (Fig. S4). Of note, in studies with these two PC cells (and nine additional pancreatic and non-PC cell lines), P-V, which inhibited their in vitro growth more potently than VPA, displayed a triple cytokinetic effect: a) inhibition of proliferation; b) block at the G_2_/M → G_1_ cell cycle transition, associated with enhanced p21 expression; and c) induction of apoptosis (Fig. S5), which was selective, sparing the normal pancreatic acinar cells (Fig. S6).

P-V was well tolerated by the mice in all studies (Fig. S1C), and showed no genotoxicity on Ames test and no acute toxicity in mice (Results S1).

### P-V inhibits STAT3 signaling in PC: Cytosolic/nuclear effect

Identifying the molecular target(s) of a new agent is an essential part of its development. Because VPA inhibits histone deacetylation, we initially examined whether P-V could also inhibit histone deacetylation. For this purpose, we measured HDAC activity in Panc-1 cells after 3 h of incubation with P-V at concentrations corresponding to 1×and 1.5×IC_50_ for cell growth. P-V inhibited HDAC activity by 23 and 29%, respectively whereas VPA at its IC_50_ inhibited it by 56%. As expected, trichostatin A strongly inhibited HDAC activity by 89% (Fig. S7A)

Because NF-κB is constitutively activated in 70% of human pancreatic cancers and in human pancreatic cell lines [23]–[25], we also examined if P-V inhibited its activation. As shown in Fig. S7B, P-V only modestly inhibited NF-κB activation in BxPC-3 cells.

Using an antibody microarray analysis (Kinexus, Vancouver, Canada), we identified STAT3 as a potential target of P-V. Following a 2 h treatment of human PC cells with P-V 15 µM, the most pronounced effect was the inhibition of STAT3 phosphorylation. Given the above, we examined the STAT3 pathway further.

**Figure 2:**
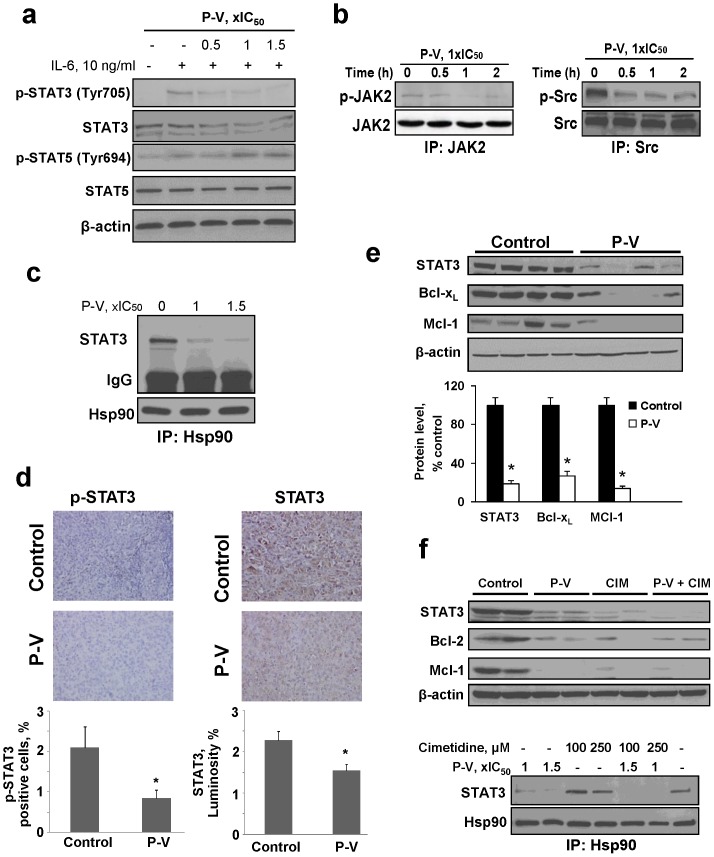
P-V inhibits STAT3 signaling in vitro and in vivo. (**A**) Immunoblots of STAT3, phosphorylated STAT3 (p-STAT3), STAT5 and p-STAT5 from BxPC-3 cells treated with P-V for 1 h and stimulated with IL-6 for 30 min. (**B**) JAK2 and Src were immunoprecipitated from control and P-V-treated MIA PaCa-2 cells and the precipitates were immunoblotted. (**C**) Hsp90 was immunoprecipitated from control and P-V-treated BxPC-3 cells and the precipitate was immunoblotted for STAT3. IgG  =  Loading control. (**D**) Immunostaining for STAT3 and phosphorylated STAT3 (p-STAT3) expression on tissue sections of MIA PaCa-2 orthotopic tumors from control and P-V-treated mice (x40). Results are expressed as percent of positive cells for p-STAT3 and luminosity index per 40×field for STAT3. Values are mean±SEM (n = 7); *p<*0.05 vs*. control. (**E**) Protein lysates from BxPC-3 xenografts were immunoblotted for STAT3, Bcl-x_L_ and Mcl-1 proteins. Each lane represents a different tumor sample. Loading control: β-actin. Bands were quantified and results expressed as percent control for each protein. Values are mean±SEM (n = 7); *p<*0.01 vs*. control. (**F**) (*Upper*) Cimetidine (CIM) enhances P-V's inhibitory effect on STAT3 in vivo. Protein lysates from MIA PaCa-2 xenografts were analyzed for STAT3, Bcl-2 and Mcl-1 proteins by immunoblotting. Each lane represents a different tumor sample. Loading control: β-actin. (*Lower*) CIM enhances P-V's disruption of the STAT3-Hsp90 association. Hsp90 was immunoprecipitated from MIA PaCa-2 cells treated with or without P-V, CIM or both. After immunoprecipitation, immunoblotting for STAT3 was performed.

In PC cell lines, P-V inhibited both constitutive and IL-6-stimulated STAT3 activation, (but not STAT5; Fig 2A), decreasing STAT3 phosphorylation and blocking its binding to DNA. As a result, the expression of STAT3-dependent proteins such as Bcl-x_L_, Mcl-1 and survivin, which contribute to resistance to apoptosis was suppressed (Fig. 2A–B; Fig. S8). P-V also inhibited events upstream of STAT3 phosphorylation, including both JAK2 and Src phosphorylation (Fig. 2B). As shown in Fig. 2C, P-V disrupted the association between STAT3 and Hsp90 without affecting its levels (Fig. S9); the chaperone protein Hsp90 facilitates STAT3 phosphorylation by optimizing its conformation for phosphorylation [26]. Immunohistochemical studies of orthotopic PC xenografts revealed that P-V inhibited p-STAT3 and total STAT3 expression in vivo as well (Fig. 2D). In mice with BxPC-3 xenografts treated with P-V, STAT3 expression was reduced by 81%, compared to control (p<0.01; Fig. S10). Immunoblots of protein lysates from xenografts confirmed these observations. P-V suppressed the expression of STAT3-dependent proteins Bcl-x_L_ and Mcl-1 in these tumors (Fig. 2E).

Combined with cimetidine, P-V also suppressed the expression of STAT3, Bcl-2 and Mcl-1 in heterotopic MIA PaCa-2 xenografts (Fig. 2F). Of interest, cimetidine enhanced the ability of P-V to disrupt the Hsp90-STAT3 association in MIA PaCa-2 cells (Fig. 2F). Furthermore, pretreatment of PC cells with ranitidine, another H-2 blocker, failed to modulate the growth inhibitory effect of cimetidine (Fig. S11), suggesting that cimetidine may not act exclusively by inhibiting H-2 receptors.

### P-V inhibits STAT3 signaling in PC: Mitochondrial effect

**Figure 3:**
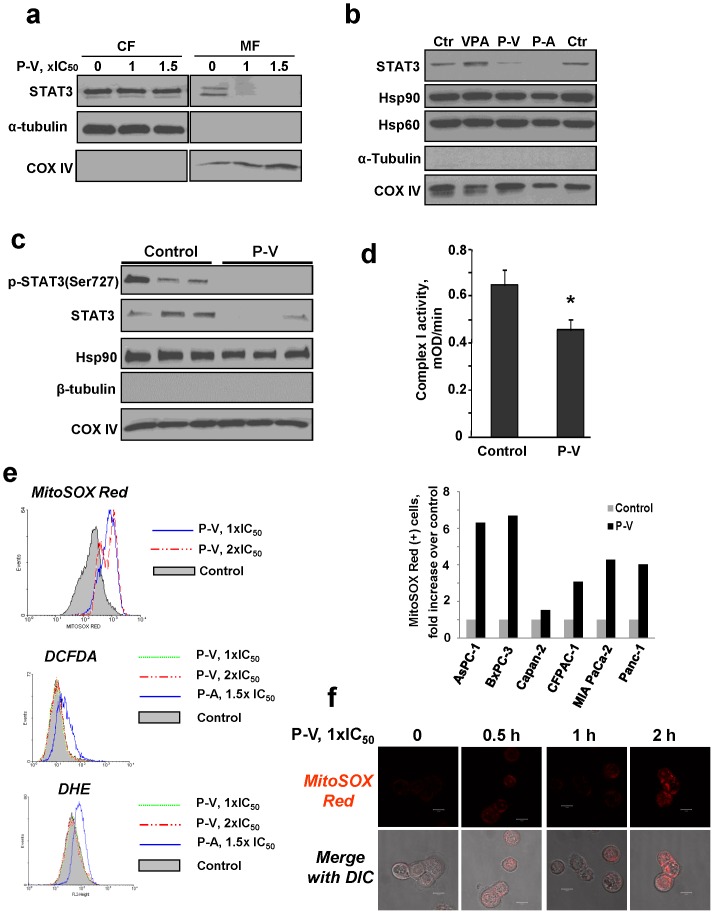
P-V inhibits STAT3 mitochondrial localization and selectively induces mitochondrial ROS in PC cells. (**A**) Immunoblots for STAT3, α-tubulin or COX IV in cytosolic (CF) and mitochondrial (MF) fractions from MIA PaCa-2 cells treated with P-V for 5 h. (**B**) Immunoblots for STAT3, Hsp90, Hsp60, α-tubulin and COX IV in mitochondrial fractions from MIA PaCa-2 cells treated with P-V, VPA or phospho-aspirin (P-A) for 3 h. (**C**) Immunoblots for p-STAT3^Ser727^, STAT3 and Hsp90 in mitochondrial fractions isolated from MIA PaCa-2 xenografts from control or 150 mg/kg P-V-treated mice. β-tubulin  =  cytosolic cross-contamination control; COX IV  =  mitochondrial loading control. Each lane corresponds to a different tumor sample. (**D**) P-V decreases the activity of the mitochondrial electron transfer chain complex I (NADPH dehydrogenase). The activity of the mitochondrial complex I was measured as described in Methods. Values are mean±SEM (n = 3); *p<*0.05 vs*. control. (**E**) MitoSOX Red, DCFDA and DHE fluorescence was measured by flow cytometry in BxPC-3 cells treated with P-V for 1 h. As positive controls, we treated cells with phospho-aspirin (P-A) for 1 h, which induces both DCFDA and DHE [13]. *Right panel*: Mitochondrial O_2_^−^ levels in a panel of six PC cells treated with P-V 1.5×IC_50_ for 1 h. (**F**) BxPC-3 cells were treated with P-V for up to 2 h, followed by addition of the MitoSOX Red probe and cells were analyzed by confocal microscopy (x40).

In the mitochondria, STAT3 supports Ras-dependent malignant transformation and is required for optimal function of the electron transport chain [4], [5]. In PC cells, P-V decreased the levels of STAT3 in the mitochondria without affecting its cytoplasmic levels (Fig. 3A and Fig. S12). Interestingly, VPA had no effect on mitochondrial STAT3 levels. In contrast, phospho-aspirin, which shares with P-V the same aromatic linker [13], reduced mitochondrial STAT3 levels (Fig. 3B), suggesting that the linker moiety may participate in this effect. None of these three compounds affected the mitochondrial levels of Hsp90 and Hsp60 proteins, both imported into the mitochondria, indicating that the changes in STAT3 levels were not due to a generalized suppression of protein transport into the mitochondria.

Nuclear-encoded proteins are imported into mitochondria mainly through the translocases of the mitochondrial outer membrane (TOM) complex. TOM20, a component of this complex, is essential to the specificity of this process [27]. P-V impaired the association between STAT3-TOM20, shown by immunoprecipitating mitochondrial fractions with an anti-TOM20 antibody and immunoblotting for STAT3 (Fig. S13A). This finding suggests that P-V reduces the mitochondrial import of STAT3. This was confirmed by a mitochondria import assay. ^35^S-methionine labeled STAT3, generated by *in vitro* translating *stat3*, was treated with or without P-V prior to its interaction with intact mitochondria. P-V reduced the level of ^35^S-STAT3 in the mitochondria, establishing its ability to inhibit the mitochondrial transport of STAT3 (Fig. S13B). That P-V inhibits mitochondrial STAT3 was confirmed in vivo: p-STAT3^Ser727^ and STAT3 levels were decreased in mitochondria isolated from PC xenografts of P-V-treated mice, while those of the Hsp90 protein remained unaffected (Fig. 3C).

**Figure 4:**
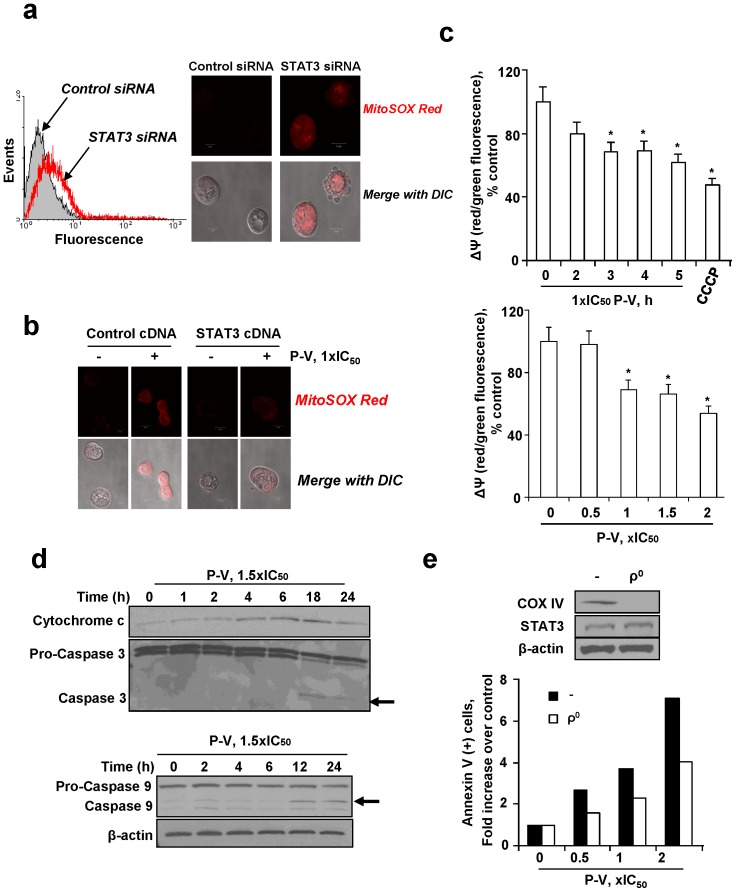
P-V blocks the mitochondrial membrane potential and induces mitochondrial cell death: The role of mitochondrial STAT3. (**A**) Knocking-down STAT3 increases mitochondrial O_2_^−^ levels in BxPC-3 cells. Control-siRNA and STAT3-siRNA cells were preloaded with MitoSOX Red, and mitochondrial O_2_^−^ was determined by flow cytometry (*left*) or confocal microscopy (*right*; x40). (**B**) STAT3 overexpression reduces the increase in mitochondrial O_2_^−^ induced by P-V. BxPC-3 cells were transfected with a STAT3-expressing plasmid or a control plasmid (empty vector) for 48 h, and then treated with P-V for 4 h followed by addition of the probe MitoSOX Red. Cells were then examined by confocal microscopy (x40). (**C**) P-V collapses the mitochondrial membrane potential (ΔΨm) in a time- (*top*) and concentration-dependent (*bottom*) manner. Fluorescence histograms were quantified and results are shown as mean±SEM; *p<*0.05 vs*. control. (**D**) Immunoblots for cytochrome c, pro-caspases and caspases 9 and 3 in cytosolic protein extracts from BxPC-3 cells treated with P-V. (**E**) BxPC-3 mitochondria-less (ρ^0^) cells are markedly more resistant to P-V-induced apoptosis. *Top*: Immunoblots for COX IV and STAT3 in parental (−) and ρ^0^ BxPC-3 cells. Loading control: β-actin.

STAT3 optimizes the function of the electron transport chain,[5] the main generator of ROS. In MIA PaCa-2 cells, P-V decreased by 29% (p<0.05) the activity of mitochondrial complex I (Fig. 3D), affecting ROS production. Using MitoSOX Red, a molecular probe specific for mitochondrial superoxide anion (O_2_^−^), we showed that P-V increased O_2_^−^ levels in mitochondria in six PC cell lines (Fig. 3E-F). This effect was mitochondria-specific, as P-V failed to induce other cellular ROS, determined using DCFDA (detects multiple ROS species) and DHE (probes for total cellular O_2_^−^). As positive controls, we used phospho-aspirin, previously shown to induce both DCFDA and DHE [13]. Of note, VPA had no effect on ROS, including mitochondrial O_2_^−^ (Fig. S14). As combination partners, P-V and cimetidine synergized to increase mitochondrial O_2_^−^ levels. P-V and cimetidine each alone increased the proportion of MitoSOX Red(+) PC cells 3.7- and 0.1-fold, respectively, and 5.6-fold in combination (Fig. S15A). The effect of P-V on STAT3 was pivotal to the generation of mitochondrial O_2_^−^. This notion is supported by the finding that when STAT3 was knocked down, the levels of mitochondrial O_2_^−^ increased (Fig. 4A), indicating that STAT3 inhibited the production of O_2_^−^ by the mitochondria. In contrast, when we overexpressed STAT3, which included its overexpression in mitochondria, the increase in mitochondria O_2_^−^ levels in response to P-V was abrogated (Fig. 4B).

A consequence of the increased mitochondrial ROS levels was the collapse of the mitochondrial membrane potential (ΔΨm). Treatment of PC cells with P-V collapsed their ΔΨm, evidenced by the decreased red/green fluorescent ratio of cells preloaded with the probe JC-1 (Fig. 4C). The collapse of ΔΨm activated the intrinsic apoptotic pathway, manifested by the release of cytochrome c to the cytosol (starting 4 h after treatment initiation); cleavage of procaspase 9 (starting at 12 h); and activation of caspase 3 (starting at 18 h) (Fig. 4D). The synergy between P-V and cimetidine was also evident in the induction of apoptosis. After 24 h of treatment with P-V/cimetidine, the fold-increase of annexinV(+) cells was 2.6, compared to 1.1 for P-V and none for cimetidine alone (Fig. S15B).

These findings suggest that mitochondria may mediate the PC cell killing effect of P-V. Consequently, we generated mitochondria-less (ρ^0^) BxPC-3 cells (the absence of the mitochondrial protein cytochrome c oxidase subunit IV (COX IV) from the ρ^0^ cells confirmed their lack of mitochondria). Compared to parental cells, ρ^0^ cells were 43% more resistant to P-V-induced apoptosis, establishing that mitochondria mediate in part the induction of cell death by P-V (Fig. 4E).

### Overexpression of mitochondrial STAT3 abrogates the anticancer effect of P-V

To confirm that STAT3 is a key target of P-V, we generated MIA PaCa-2 cells stably overexpressing STAT3 (STAT3*^high^*), using as controls their parent cells that have basal levels of STAT3 (STAT3*^normal^*). STAT3 overexpression abrogated the growth inhibitory and proapoptotic effects of P-V in vitro (Fig. S16). For example, the induction of apoptosis by P-V in STAT3*^high^* cells was less than half of that in STAT3*^normal^* cells (2.2 vs. 5.2 fold increase of annexin V(+) cells after 24 h of treatment; Fig. S16B). Similarly, overexpression of STAT3 reduced by half the induction of mitochondrial O_2_^−^ levels by P-V (6.1-fold increase of MitoSOX Red(+) cells in STAT3*^high^* cells vs. 12.6-fold increase in STAT3*^normal^* cells; Fig. S16C). The overexpression of STAT3 in mitochondria of STAT3*^high^* cells was confirmed by immunoblotting.

**Figure 5:**
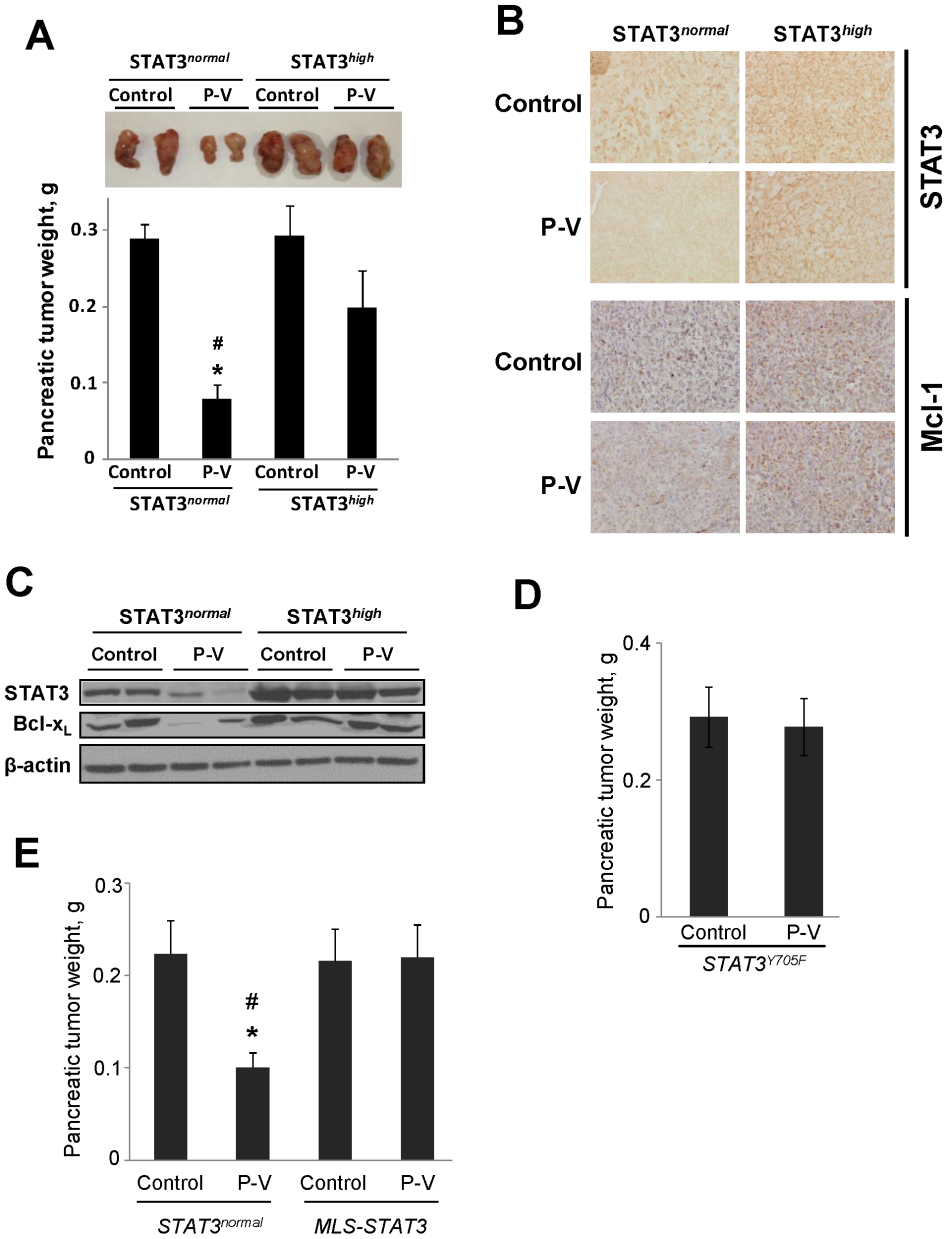
Mitochondrial STAT3 overexpression abrogates the anticancer effect of P-V in vivo. (**A**) MIA PaCa-2 cells with basal (STAT3*^normal^*) or overexpressed STAT3 (STAT3*^high^*) levels were orthotopically implanted in nude mice, which were then treated without (control) or with P-V 150 mg/kg for 18 days. *Top*: Images of representative pancreatic tumors. *Bottom*: Pancreatic tumor weight (mean ± SEM). *p<*0.01 vs*. control; ^#^ *p*<*0.01 vs*. STAT3*^high^* P-V-treated group. (**B**) STAT3 and Mcl-1 expression in STAT3*^normal^* and STAT3*^high^* MIA PaCa-2 orthotopic tumor tissue sections from control and P-V-treated mice (x20). (**C**) Immunoblots for STAT3 and Bcl-x_L_ in orthotopic tumor samples. Each lane represents a different tumor sample. Loading control: β-actin. (**D**) MIA PaCa-2 cells with overexpressed STAT3 Y705F mutant (STAT3*^Y705F^*) levels were orthotopically implanted in nude mice, which were then treated without (control) or with P-V 150 mg/kg for 18 days. Pancreatic tumor weight (mean±SEM). (**E**) AsPC-1 cells with basal (STAT3*^normal^*) or overexpressed mitochondria-targeted STAT3 (MLS-STAT3) levels were orthotopically implanted in nude mice, which were then treated without (control) or with P-V 150 mg/kg for 21 days. Pancreatic tumor weight (mean±SEM). *p<*0.01 vs*. control; ^#^ *p*<*0.01 vs*. MLS-STAT3 P-V-treated group.

To assess these findings in vivo, we implanted orthotopically into nude mice STAT3*^high^* or STAT3*^normal^* cells. After 18 days of treatment, P-V failed to affect the growth of STAT3*^high^* tumors, in contrast to 72% inhibition of STAT3*^normal^* tumors (p<0.001; Fig. 5A). P-V failed to suppress the levels of STAT3 or its dependent proteins Bcl-x_L_, Mcl-1 and Bcl-2 in STAT3*^high^* tumors, whereas it did suppress them all in STAT3*^normal^* tumors (Fig. 5B–C and S17).

To confirm that mitochondrial STAT3 is a significant target of P-V, we generated MIA PaCa-2 cells stably overexpressing STAT3*^Y705F^* mutant [16], which retains mitochondrial function but lacks nuclear transcriptional activity [5]. We implanted these cells orthotopically into nude mice, and treated them with or without P-V for 18 days. At sacrifice, P-V failed to affect the growth of STAT3*^Y705F^* tumors, whose weight was comparable to that of control mice (Fig. 5D).

To further examine the role of mitochondrial STAT3 as a critical target of P-V, we generated AsPC-1 cells stably overexpressing mitochondrial-targeted STAT3 (MLS-STAT3) [17], using as controls their parent cells (STAT3*^normal^*). We implanted orthotopically into nude mice the cells overexpressing MLS-STAT3 or STAT3*^normal^* cells, and treated them with or without P-V for 21 days. At sacrifice, P-V failed to affect the growth of MLS-STAT3 tumors, while it inhibited by 55% STAT3*^normal^* tumors (p<0.01; Fig. 5E). Furthermore, P-V reduced mitochondrial STAT3 levels in STAT3*^normal^* cells, but not in MLS-STAT3 cells (Fig. S18).

## Discussion

PC, a devastating malignancy with unsatisfactory treatment options, is in pressing need for new agents. We approached this need by developing a novel agent that strongly inhibits PC while it apparently lacks toxicity. Based on our findings from human PC xenografts in mice, the inhibition of PC growth by P-V appears to be: very strong, ranging between 60% and 97% and being twice as strong as that of its parent compound VPA; independent of the *Kras* status of PC cells; and synergistic with cimetidine. Interestingly, P-V is effective when given either under prevention or treatment protocols.

**Figure 6:**
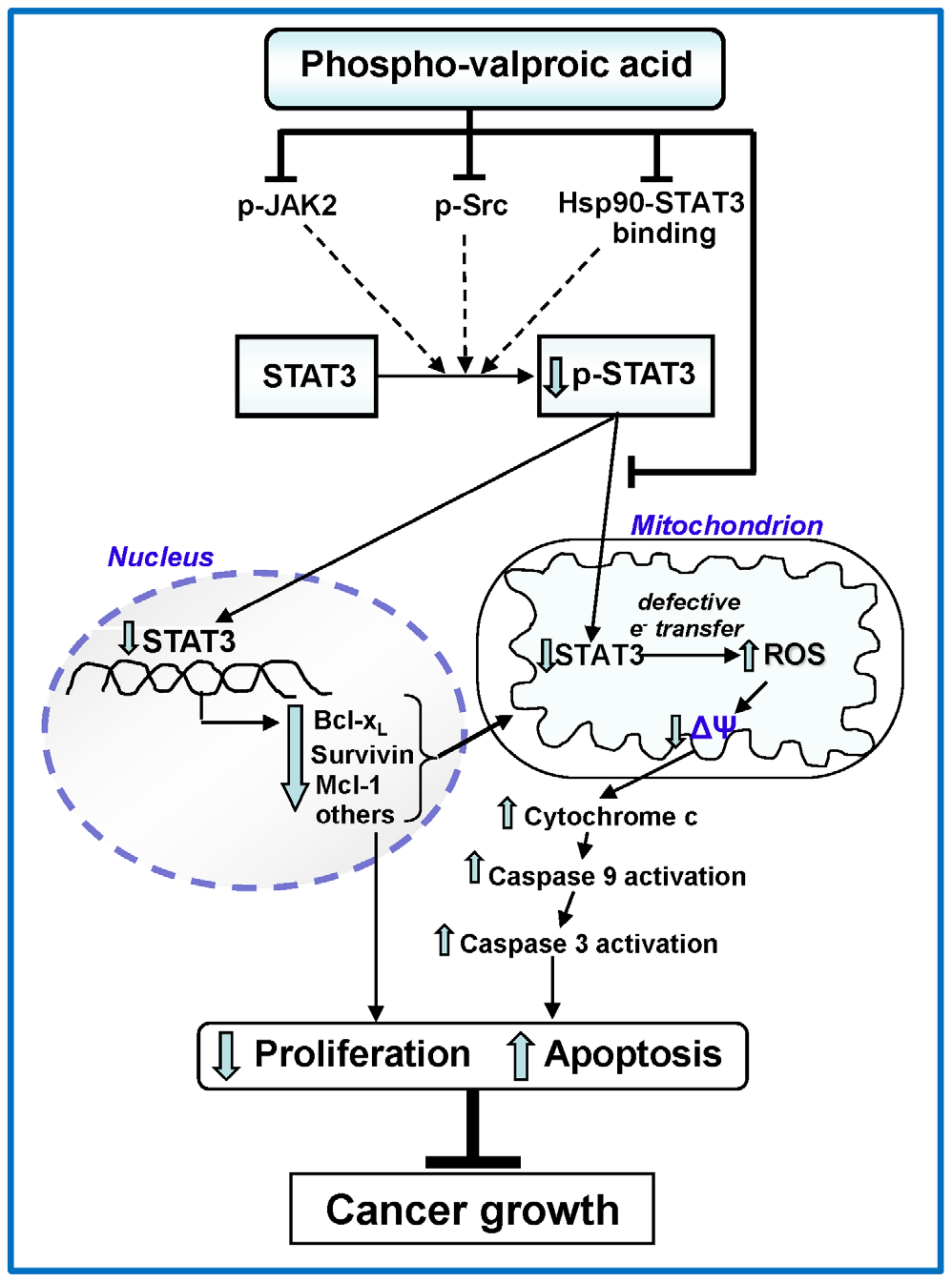
Proposed mechanism of action of P-V.

STAT3, which plays an essential role in the initiation and progression of PC [2], [3] and in the induction of resistance to apoptosis, is the key molecular target of P-V [7]. The inhibition of STAT3, but not STAT5, by P-V is consequential for the fate of the PC cell. P-V has two distinct effects: it inhibits the activating phosphorylation of STAT3, and blocks the entry of STAT3 into the mitochondria where it exerts a profound metabolic effect (Fig. 6). P-V suppressed both the phosphorylation of STAT3 in vitro and in vivo and its expression in vivo. When STAT3 levels were assayed in the cytoplasm and mitochondria, the cytosolic levels of STAT3 were not suppressed in contrast to those in mitochondria. This finding, which may seem to vary in part from previous ones assessing total cellular STAT3 levels, may be explained by differences in the duration of exposure to the drug or perhaps a differential sensitivity between the cytosolic and mitochondrial fractions. The inhibition of STAT3 phosphorylation by P-V is likely the result of a triple effect: a) inhibition of JAK2 phosphorylation; b) inhibition of Src activation; and c) disruption of the Hsp90-STAT3 association, which optimizes the conformation of STAT3 for phosphorylation [26]. Of note, JAK2 and Src are overexpressed in human PC, making this effect therapeutically plausible [6]. The suppressed activation of STAT3 leads to a decreased expression of STAT3-regulated proteins, including antiapoptotic gene products, such as Mcl-1, survivin, bcl-2, and bcl-xL in PC cells and xenografts. The downregulation of these anti-apoptotic proteins likely contributes to P-V's proapoptotic effect that underlies its ultimate anticancer effect.

Mitochondrial STAT3 proved an important molecular target of P-V, which blocked its import through a mechanism as yet not entirely clear. Our data show that at the very least P-V suppresses the interaction of STAT3 with Hsp90 and TOM20, both of which normally interact physically with STAT3, facilitating its entry into the mitochondria [26], [28]. It is conceivable that P-V chemically modifies STAT3 (and/or Hsp90 and TOM20), sterically inhibiting their physical interaction and thereby blocking the mitochondrial import of STAT3. This notion is based on our finding that phosphoaspirin (MDC-43), but not VPA, reduces STAT3 mitochondrial levels; phospho-aspirin shares with P-V the same benzyl linker moiety [13], which is chemically active and could covalently modify STAT3 at its cystein thiol groups [29]. Interestingly, Cys^468^ of STAT3, a therapeutic target, is known to be susceptible to alkylation [30].

The inhibition of STAT3 import into the mitochondria by P-V had serious implications since STAT3 regulates cellular respiration in mitochondria [5]. The decreased STAT3 levels were directly responsible for the enhanced generation of O_2_^−^ selectively by the mitochondria, an effect accounted for by disruptions in the electron transfer chain of the mitochondria, which involves at least complex I. The resultant oxidative stress triggered the intrinsic apoptotic cascade of the PC cells, manifested by collapsed ΔΨm, release of cytochrome c and the downstream activation of execution caspases. In addition, the lack of STAT3 could have a direct effect on ΔΨm, as STAT3 is known to inhibit the mitochondrial permeability transition pore opening [28]. In agreement with our findings, STAT3 disruption was reported to decrease mitochondrial function and increase oxidative stress in astrocytes [31] and cardiomyocytes [17]. The centrality of STAT3 and mitochondria in the induction of apoptosis by P-V was documented by the finding that both mitochondrial STAT3 overexpression and the ablation of mitochondria (ρ^0^ cells) abrogated in part the anticancer effect of P-V.

In conclusion, P-V, the novel inhibitor of mitochondrial STAT3, either alone or in combination with cimetidine is an effective anticancer agent in preclinical models of PC. STAT3 is the dominant molecular target of P-V, which merits evaluation as a promising candidate drug for PC.

## Supporting Information

Figure S1 **P-V inhibits the growth of human pancreatic cancer in an orthotopic model.**
(TIF)

Figure S2 **Anticancer agents synergize with P-V to inhibit pancreatic cancer cell growth.**
(TIF)

Figure S3 **5-FU fails to synergize with P-V in vivo.**
(TIF)

Figure S4 **Apoptosis and cell proliferation expression in BxPC-3 tumors from control and P-V-treated mice.**
(TIF)

Figure S5 **The cell kinetic effect of P-V on pancreatic cancer cells.**
(TIF)

Figure S6 **P-V selectively induces apoptosis in pancreatic tumors.**
(TIF)

Figure S7 **Cell signaling effects of P-V in human pancreatic cancer cells.**
(TIF)

Figure S8 **P-V inhibits the STAT3 signaling pathway in human pancreatic cancer cells.**
(TIF)

Figure S9 **P-V does not affect Hsp90 levels in human pancreatic cancer cells.**
(TIF)

Figure S10 **P-V suppresses STAT3 expression in human pancreatic cancer xenografts.**
(TIF)

Figure S11 **Pretreatment of human pancreatic cancer cells with ranitidine, another H-2 blocker, fails to modulate the growth inhibitory effects of cimetidine.**
(TIF)

Figure S12 **The presence of STAT3 in mitochondria.**
(TIF)

Figure S13 **P-V inhibits TOM20-STAT3 association and prevents STAT3 mitochondrial localization.**
(TIF)

Figure S14 **VPA fails to induce ROS in human pancreatic cancer cells.**
(TIF)

Figure S15 **P-V synergizes with cimetidine to increase mitochondrial ROS and induce apoptosis in human pancreatic cancer cells.**
(TIF)

Figure S16 **STAT3 overexpression abrogates cell growth inhibition by P-V.**
(TIF)

Figure S17 **STAT3 modulates Bcl-2 expression in pancreatic cancer xenografts.**
(TIF)

Figure S18 **Immunoblots for STAT3 or COX IV in mitochondrial fractions.**
(TIF)

Methods S1 **Supplemental Methods.**
(PDF)

Results S1 **Supplemental Results.**
(PDF)
