# Supplementary figures and images for: Targeting Mitochondrial STAT3 with the Novel Phospho-Valproic Acid (MDC-1112) Inhibits Pancreatic Cancer Growth in Mice
Source: PLoS One. 2013 May 1;8(5):e61532. doi: 10.1371/journal.pone.0061532 (PMC3641121; doi:10.1371/journal.pone.0061532)

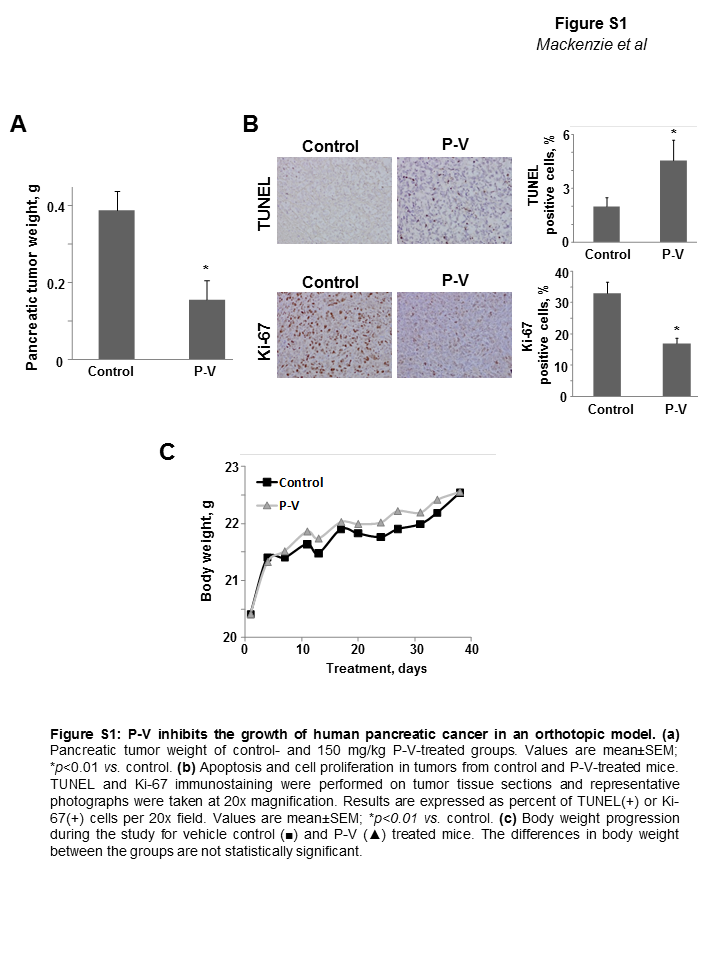

Supplement: Figure S1 — P-V inhibits the growth of human pancreatic cancer in an orthotopic model. (TIF) [file pone.0061532.s001.tif]

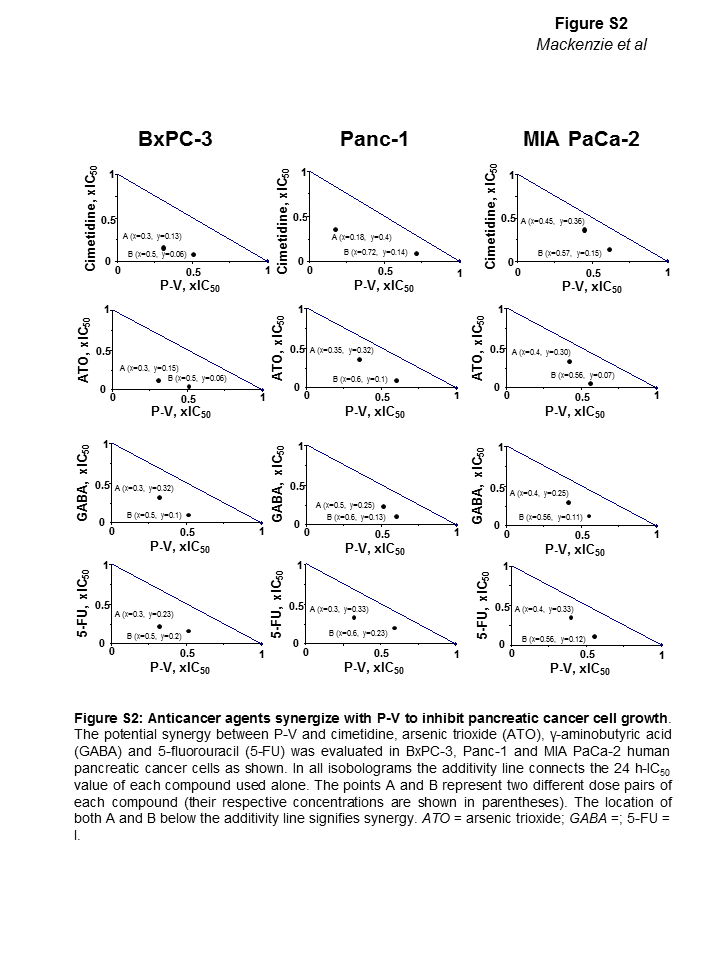

Supplement: Figure S2 — Anticancer agents synergize with P-V to inhibit pancreatic cancer cell growth. (TIF) [file pone.0061532.s002.tif]

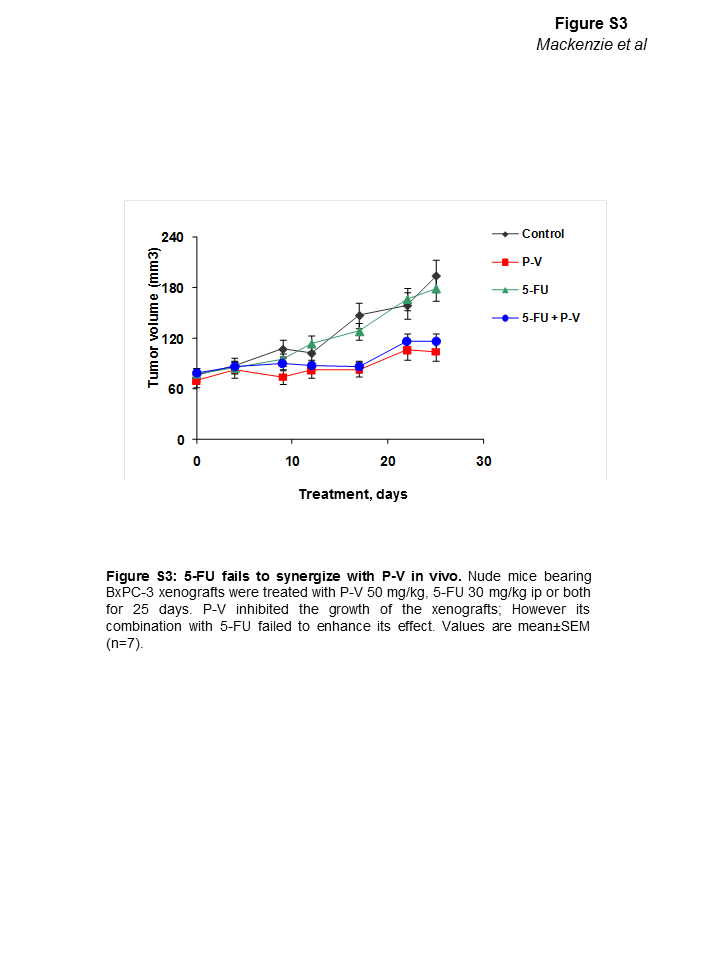

Supplement: Figure S3 — 5-FU fails to synergize with P-V in vivo. (TIF) [file pone.0061532.s003.tif]

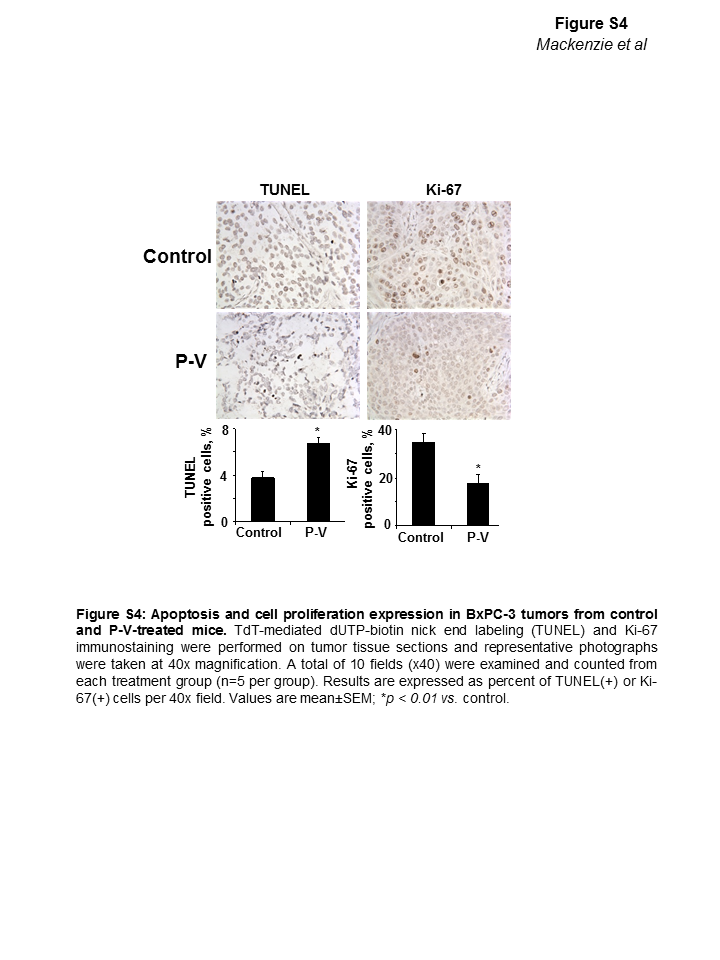

Supplement: Figure S4 — Apoptosis and cell proliferation expression in BxPC-3 tumors from control and P-V-treated mice. (TIF) [file pone.0061532.s004.tif]

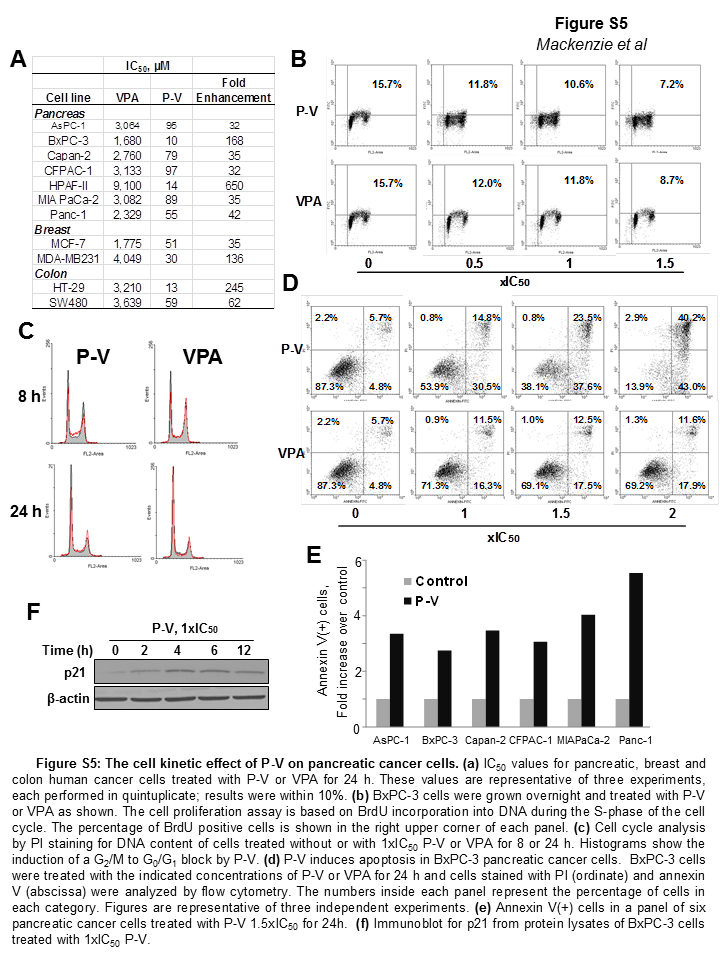

Supplement: Figure S5 — The cell kinetic effect of P-V on pancreatic cancer cells. (TIF) [file pone.0061532.s005.tif]

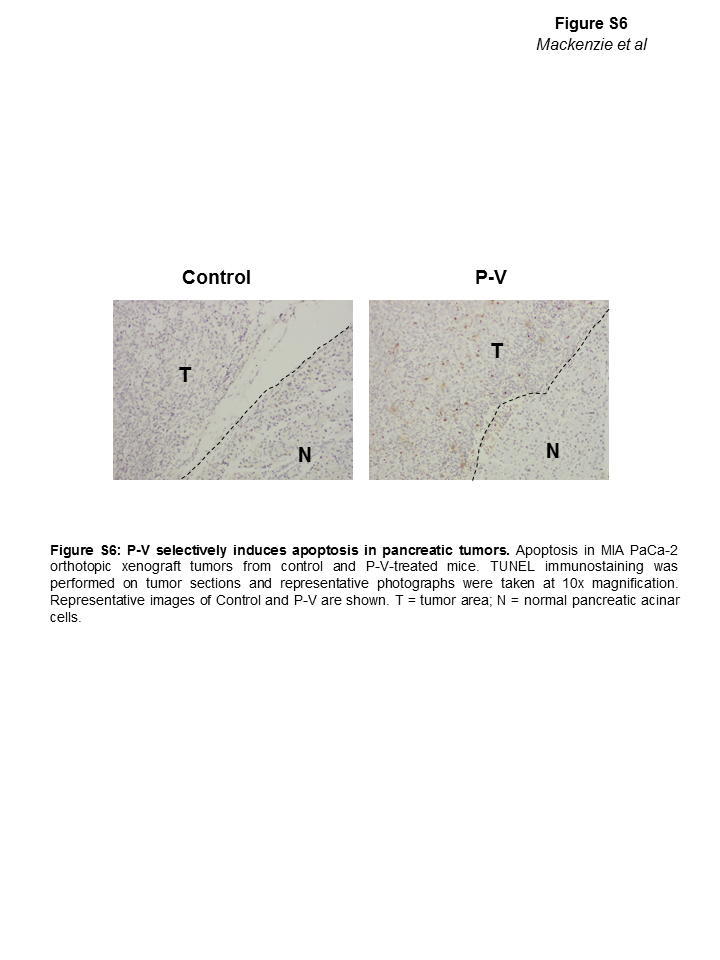

Supplement: Figure S6 — P-V selectively induces apoptosis in pancreatic tumors. (TIF) [file pone.0061532.s006.tif]

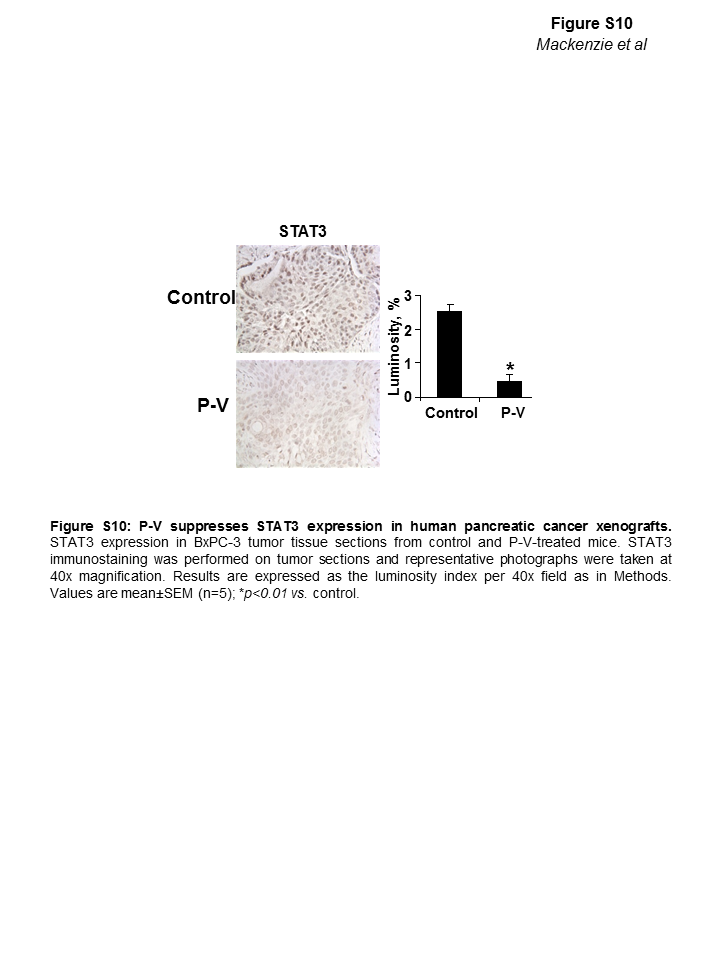

Supplement: Figure S10 — P-V suppresses STAT3 expression in human pancreatic cancer xenografts. (TIF) [file pone.0061532.s010.tif]

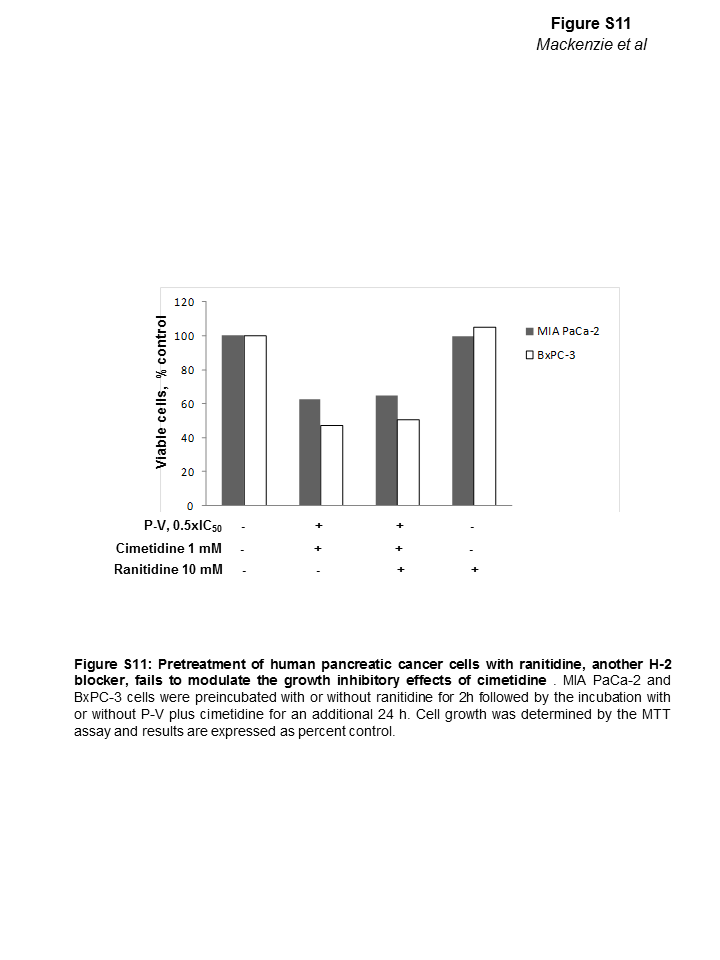

Supplement: Figure S11 — Pretreatment of human pancreatic cancer cells with ranitidine, another H-2 blocker, fails to modulate the growth inhibitory effects of cimetidine. (TIF) [file pone.0061532.s011.tif]

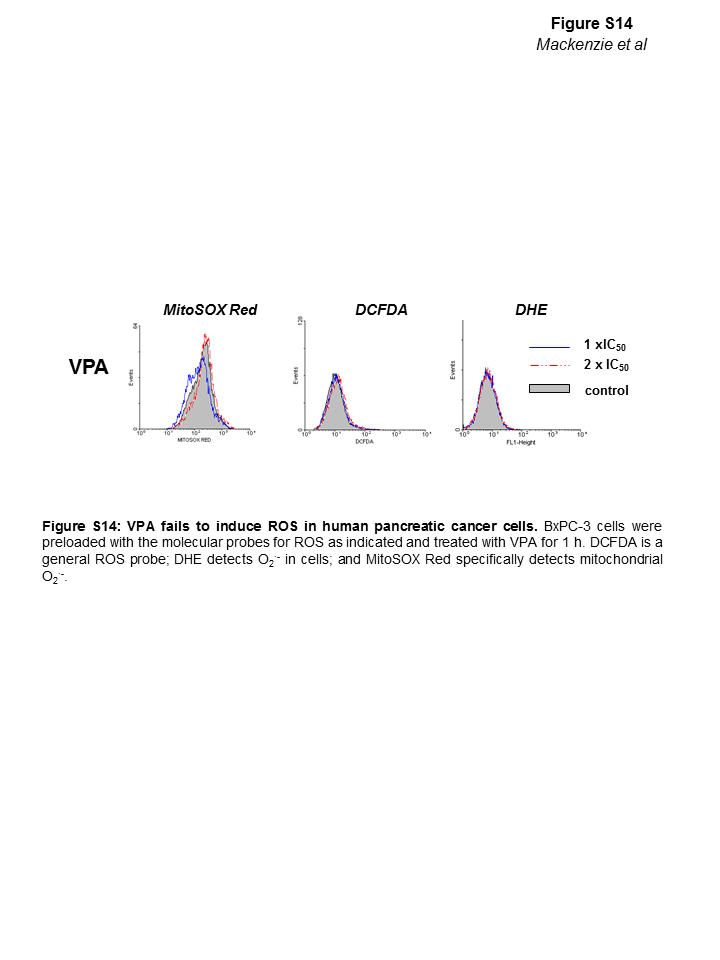

Supplement: Figure S14 — VPA fails to induce ROS in human pancreatic cancer cells. (TIF) [file pone.0061532.s014.tif]

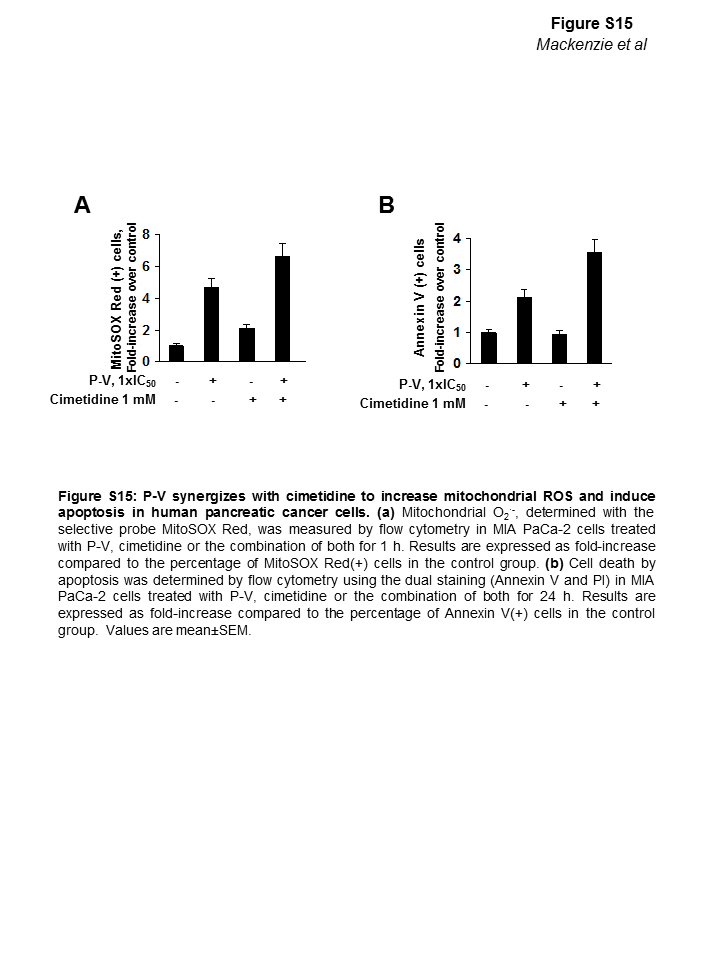

Supplement: Figure S15 — P-V synergizes with cimetidine to increase mitochondrial ROS and induce apoptosis in human pancreatic cancer cells. (TIF) [file pone.0061532.s015.tif]

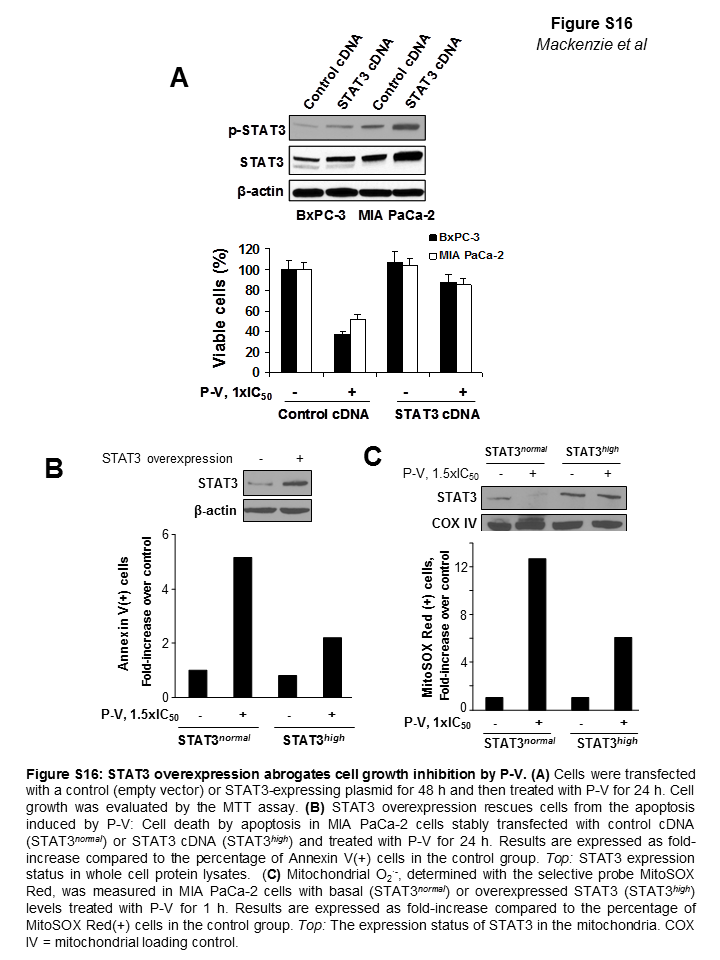

Supplement: Figure S16 — STAT3 overexpression abrogates cell growth inhibition by P-V. (TIF) [file pone.0061532.s016.tif]

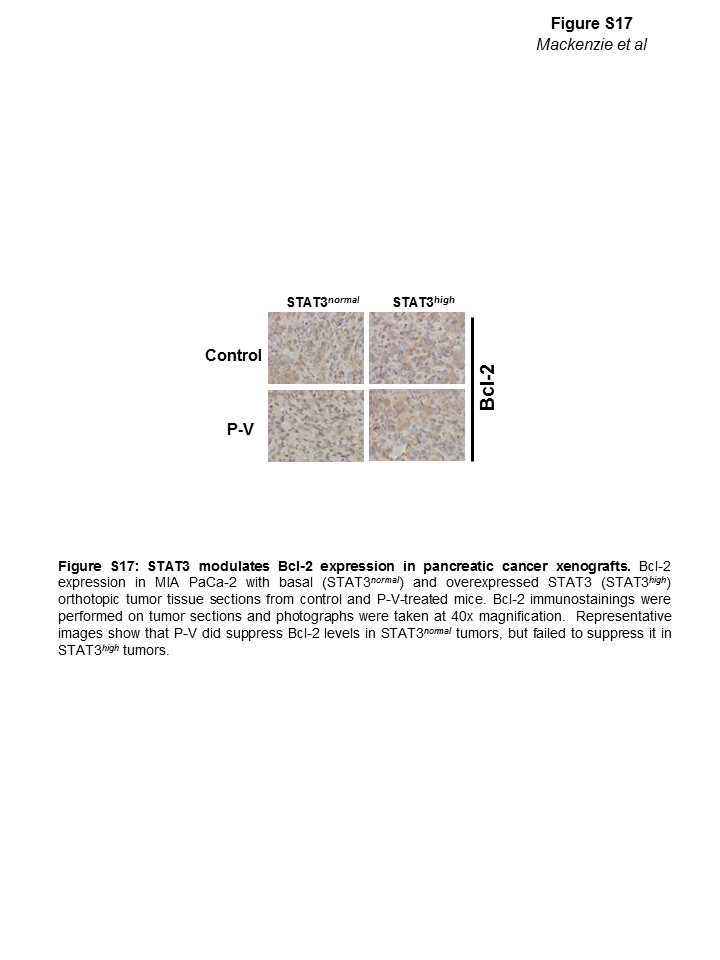

Supplement: Figure S17 — STAT3 modulates Bcl-2 expression in pancreatic cancer xenografts. (TIF) [file pone.0061532.s017.tif]
