# Supplementary material for: Targeting Mitochondrial STAT3 with the Novel Phospho-Valproic Acid (MDC-1112) Inhibits Pancreatic Cancer Growth in Mice
Source: PLoS One. 2013 May 1;8(5):e61532. doi: 10.1371/journal.pone.0061532 (PMC3641121; doi:10.1371/journal.pone.0061532)
Supplement: Methods S1 — Supplemental Methods. (PDF) [file pone.0061532.s019.pdf]

Supplemental Material

METHODS

Mitochondrial Import Assay

The *in vitro* mitochondrial import assay was performed as described elsewhere with minor modifications [1]. Briefly, cell-free translation of human recombinant STAT3 proteins was performed using the TNT-coupled transcription/translation rabbit reticulocyte lysate system (Promega, Madison, WI, USA), according to the manufacturer's directions. T7 Polymerase, included in the master mix (Promega, Madison, WI), and ^{35}S-methionine (Perkin-Elmer, Waltham, MA) were mixed with 1 µg of *stat3* plasmid (OriGene, Rockville, MD) containing the T7 promoter. The total reaction volume was adjusted to 50 µL with nuclease-free water. Reactions were incubated for 90 min at 30°C and then adjusted to 250 mM sucrose. Afterwards, 40 µl of the reaction were incubated for 60 min at 30°C with 100 µM P-V. Later, the control- and P-V-treated- ^{35}S-labeled recombinant STAT3 were diluted in import buffer (20 mM HEPES–KOH pH 7.5, 5 mM $MgOAc_2$, 80 mM KOAc, and 250 mM Sucrose) containing 2 mM ATP, 0.4 mM ADP and1 mM dithiothreitol. Bovine heart mitochondria (30 µg) were added to the translated proteins such that the volume of mitochondria made up 40% of the reaction volume and the exogenous protein constituted 35% of the total reaction volume. The translocation reaction was allowed to incubate for 45 min at 30°C with intermittent gentle mixing. Mitochondria were re-isolated by centrifugation, suspended in the import buffer in the presence of 50 µg/ml proteinase K for 10 min and mixed with 1 mM PMSF to terminate the proteolytic reaction. Mitochondria were collected by centrifugation, and imported proteins were separated by SDS gel electrophoresis, followed by autoradiography. In some experiments, 1 µg of STAT3 recombinant protein (non radioactive) was added to the import reaction before analysis of protein import into mitochondria.

Determination of Mitochondrial Membrane Potential

The mitochondrial membrane potential was determined by flow cytometry using the JC-1 cationic dye (5,5',6,6'-tetrachloro-1,1',3,3'- tetraethylbenzimidazolylcarbocyanine iodide; Invitrogen), as previously described [2]. Briefly, BxPC-3 cells were incubated with 1.5xIC_{50} P-V for 5 h and cells were trypsinized and washed once with PBS. The supernatant was discarded and cells were incubated with 5 µM JC-1 for 30 min at 37 °C protected from light and analyzed by flow cytometry using the FL1 (green) and FL2 (red) fluorescence.

Western Blot Analysis

Total cell fractions were obtained as previously described [3]. Aliquots of these fractions containing 25-40 µg protein were separated by reducing 10-12.5% (w/v) polyacrylamide gel electrophoresis and electroblotted to PVDF membranes. The following antibodies were used: p-STAT3 (Tyr705), p-STAT3 (Ser727), p-STAT5 (Tyr694) STAT3, STAT5, p-JAK2, JAK2, p-Src, Src, Bcl-X_L, Bcl-2, survivin, pro-caspase 9, pro-caspase 3 and COX IV from Cell Signaling Technologies (Danvers, MA); while STAT3, cytochrome c, Hsp90, Hsp60, α-tubulin and β-tubulin were from Santa Cruz Biotechnology (Santa Cruz, CA). β-actin (Sigma) was used as the loading control. After incubation, for 90 min at room temperature in the presence of the secondary antibody (HRP-conjugated; 1:5,000 dilution), the conjugates were visualized by chemiluminescence detection.

STAT3 Overexpression

Transient transfection: Cells were transiently transfected with nonspecific control plasmid (Control cDNA) or 100 nmol/L STAT3 expression plasmid (STAT3 cDNA) for 48 h using

Lipofectamine 2000 following the manufacturer's instructions (OriGene, Rockville, MD). Following transfection, cells were replated and treated with P-V.

Stable transfection: STAT3 human cDNA (OriGene, Rockville, MD) was initially transfected in 293T cells for 72h. The letiviral lysate was transfected in the presence of 8 µg/ml polybrene into the pancreatic cancer cells. Transfected cells were selected by incubating them for 2 weeks in growth media containing 0.5 mg/mL neomycin (G418). The cells that remain growing in the neomycin-containing medium have retained the STAT3 expression plasmid, which stably integrates into the genome of the targeted cells.

STAT3 Gene Silencing by Transfecting Specific Small Interfering RNA

Cells were transfected with 100 nmol/L STAT3 small interfering RNA (siRNA) or nonspecific control siRNA for 48 h using Lipofectamine 2000 (Invitrogen). At 48 h post transfection, cells were replated and treated with P-V.

Determination of apoptosis

Apoptosis was determined immunohistochemically by the terminal deoxynucleotidyl transferase-mediated deoxyuridine triphosphate-biotin nick end-labeling (TUNEL) assay [4]. *Scoring:* At least 10 fields per sample at magnification x20 were scored by an investigator blinded to the identity of the samples. We calculated the percentage of proliferating and apoptotic cells by dividing the number of labeled cells by the number of cells in each field and multiplying by 100. The luminosity index was determined using Adobe PhotoShop. By using the 'sampled colors' function, the positive colors were converted to white and the background was deleted. The histogram function was used to generate the luminosity, which was proportional to the intensity of staining. The gradient luminosities were calculated and used to obtain the 'mean value' generating the "immunohistochemistry (IHC) luminosity index".

Acute toxicity study

To evaluate the potential acute toxicity of P-V, six-week-old mice (n=5/group) were given for 3 weeks by i.p. 0, 25, 50, 100, 200, 300 or 400 mg/kg/d P-V or 400, 1000, 1500, or 2000 mg/kg/d VPA. Body weights were recorded twice weekly. Animals were sacrificed at 3 weeks and their organs were examined histologically for signs of toxicity.

ADDITIONAL REFERENCES

1. Barksdale KA, Bijur GN (2009) The basal flux of Akt in the mitochondria is mediated by heat shock protein 90. J Neurochem 108: 1289-1299.
2. Zhao W, Mackenzie GG, Murray OT, Zhang Z, Rigas B (2009) Phosphoaspirin (MDC-43), a novel benzyl ester of aspirin, inhibits the growth of human cancer cell lines more potently than aspirin: a redox-dependent effect. Carcinogenesis 30: 512-519.
3. Mackenzie GG, Queisser N, Wolfson ML, Fraga CG, Adamo AM, et al. (2008) Curcumin induces cell-arrest and apoptosis in association with the inhibition of constitutively active NF-kappaB and STAT3 pathways in Hodgkin's lymphoma cells. Int J Cancer 123: 56-65.

4. Mackenzie GG, Sun Y, Huang L, Xie G, Ouyang N, et al. (2010) Phospho-sulindac (OXT-328), a novel sulindac derivative, is safe and effective in colon cancer prevention in mice. Gastroenterology 139: 1320-1332.
