# Supplementary material for: Targeting Mitochondrial STAT3 with the Novel Phospho-Valproic Acid (MDC-1112) Inhibits Pancreatic Cancer Growth in Mice
Source: PLoS One. 2013 May 1;8(5):e61532. doi: 10.1371/journal.pone.0061532 (PMC3641121; doi:10.1371/journal.pone.0061532)
Supplement: Results S1 — Supplemental Results. (PDF) [file pone.0061532.s020.pdf]

Supplemental Material

RESULTS

P-V shows no genotoxicity or acute toxicity in mice

Given that the safety of novel chemopreventive agents is of extreme importance, we evaluated the safety of P-V by determining its genotoxicity by the Ames test and its toxicity in mice.

Lack of genotoxicity: The mutagenic potential of P-V was evaluated by measuring its ability to induce reverse mutations at selected loci of two strains of *Salmonella typhimurium* in the presence and absence of S9 activation (performed by BioReliance, Rockville MD). These studies indicated that P-V lacked genotoxicity.

Determination of the Maximum Tolerated Dose (MTD) in mice: Six-week old female BALB/c mice (n=5/group) were given by i.p. once a day 0, 25, 50, 100, 200, 300 or 400 mg/kg P-V or 400, 1000, 1500, or 2000 mg/kg VPA for 3 weeks. Animals were sacrificed at 3 weeks and their organs were examined. Animals treated with 300 and 400 mg/kg P-V or 1,500 and 2000 mg/kg VPA exhibited significant toxicity and died within 3 days after treatment initiation. All other groups showed no signs of toxicity, indicating that the MTD for P-V is at least 200 mg/kg/d and that of VPA is at least 1000 mg/kg.dfor a 3 week-treatment, doses up to 200 mg/kg P-V are safe.
